# The complete chloroplast genome sequence of *Hosta capitata* (Koidz.) Nakai (Asparagaceae)

**DOI:** 10.1080/23802359.2018.1511858

**Published:** 2018-10-08

**Authors:** Yeeun Jang, Jee Young Park, Shin-Jae Kang, Hyun-Seung Park, Hyeonah Shim, Taek Joo Lee, Jung Hwa Kang, Sang Hyun Sung, Tae-Jin Yang

**Affiliations:** a Department of Plant Science, Plant Genomics and Breeding Institute, and Research Institute of Agriculture and Life Sciences, College of Agriculture and Life Sciences, Seoul National University, Seoul, Korea;; b Hantaek Botanical Garden, Yongin, Korea;; c College of Pharmacy and Research Institute of Pharmaceutical Science, Seoul National University, Seoul, Korea

**Keywords:** *Hosta capitata* (Koidz.) Nakai, chloroplast genome, phylogenetic analysis

## Abstract

*Hosta capitata* (Koidz.) Nakai is an herbaceous perennial plant belonging to the Asparagaceae family and has become a popular ornamental plant. In this study, the chloroplast genome sequence of *H. capitata* was completed by *de novo* assembly with whole genome sequence data. The chloroplast genome of *H. capitata* is 156,416 bp in length, which is composed of a large single-copy (LSC) of 84,788 bp, a small single-copy (SSC) of 18,206 bp, and a pair of inverted repeat regions (IRa and IRb) of 26,711 bp, as four distinct parts. In total, 114 genes were identified including 80 protein-coding genes, 30 tRNA genes, and four rRNA genes. The phylogenetic analysis revealed that *H. capitata* has a close relationship with other *Hosta* species, *H. minor* and *H. ventricosa*, but is farther than the distance between them.

*Hosta capitata* (Koidz.) Nakai is an herbaceous perennial plant belonging to the Asparagaceae family, which is seen in southern Korea and southwestern Japan (Fuhita 1976; Chung et al. 1991). Like other *Hosta* plants, *H. capitata* has become a popular ornamental plant as a shade-tolerant garden plant with characteristics of ovate leaves, purple flowers, and smaller size than others (Chung et al. 1991; Ryu et al. 2002; Bożek et al. 2015). In spite of its horticultural and economic importance, there is few genetic and genomic study for breeding of this plant. In this study, we completed the complete chloroplast genome sequence assembly of *H. capitata* using whole genome shotgun sequences and analyzed its phylogenetic relationship with other Asparagaceae species to provide useful genetic information for breeding.

To complete the chloroplast genome sequence, the total genomic DNA was extracted from leaves of *H. capitata* provided by HanTaek Botanical Garden, Yongin, Korea. The extracted DNA was implemented for the whole genome sequencing with the Illumina MiSeq platform (Illumina, San Diego, CA). The high-quality paired-end reads of 1.5 Gbp were obtained and assembled using CLC genome assembler (ver. 4.6, CLC Inc, Aarhus, Denmark), following dnaLCW method (Kim et al. 2015a, 2015b). After *de novo* assembly, four sequence contigs representing chloroplast genome were selected, ordered and combined into a single draft sequence by comparing with *H. minor* (KX822777) chloroplast genome sequence as a reference (Kim et al. 2012).

The complete genome of *H. capitata* (GenBank Accession No. MH581151) was 156,416 bp long, which consisted of a large single-copy (LSC) of 84,788 bp, a small single-copy (SSC) of 18,206 bp, and a pair of inverted repeat regions (IRa and IRb) of 26,711 bp. In the genome, a total of 114 genes were identified, including 80 protein-coding genes, 30 tRNA genes, and four rRNA genes, through Ge-Seq annotation and BLAST tools (Tillich et al. 2017).

After multiple alignment of the chloroplast genome of *H. capitata* with those of 11 species belonging to the Asparagaceae family and *Panax ginseng* as an outgroup using MAFFT 7.0 (Katoh and Standley 2013), phylogenetic analysis was carried out based on the aligned sequences for a neighbor-joining tree by maximum composite likelihood model with 1000 of bootstrap replications of MEGA 7.0 (Kumar et al. 2016) (Figure 1). The phylogenetic tree showed that the Agavoideae and Nolinoideae subfamilies were divided and Agavoideae was further subgrouped into three genera, *Yucca*, *Agave*, and *Hosta*, respectively. To be specific, *H. capitata* was located together with *H. minor* and *H. ventricosa*, but is farther than the distance between the two *Hosta* plants.

**Figure 1.**
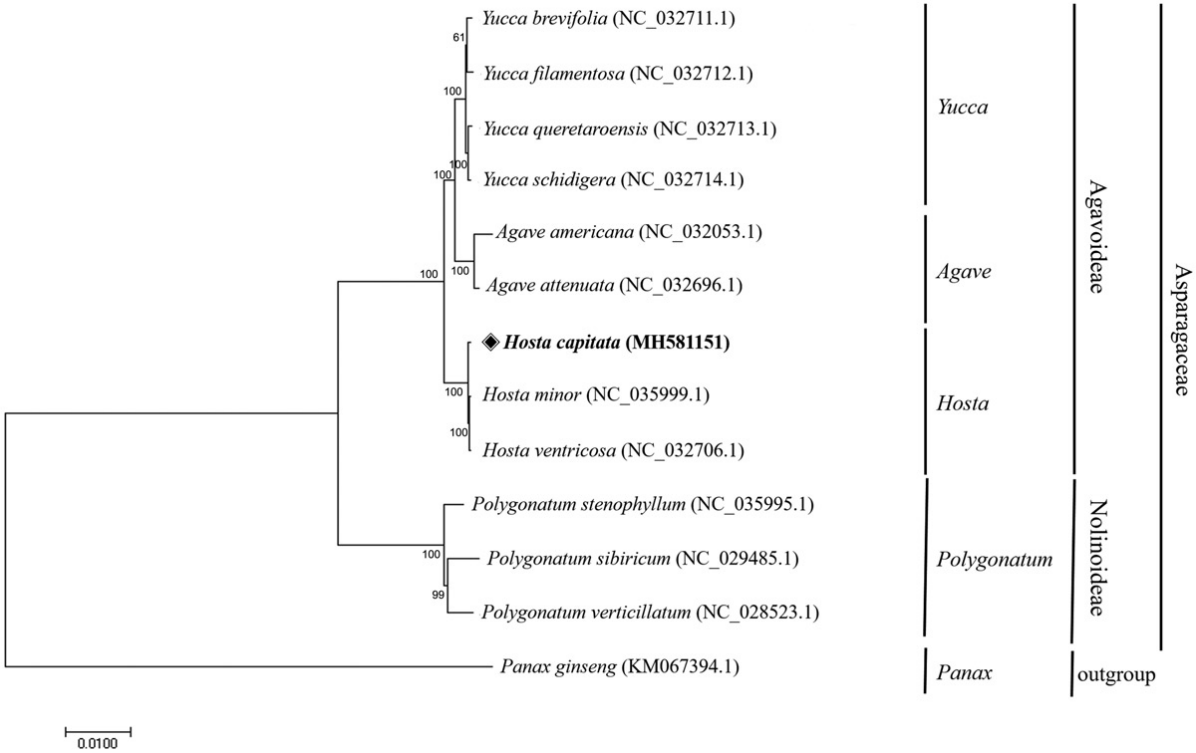
The phylogenetic tree was constructed using chloroplast genome sequences of 12 species belonging to the Asparagaceae family and *Panax ginseng* as an outgroup. On the MEGA 7.0 software, a neighbor-joining tree was constructed using maximum composite likelihood model with 1000 of bootstrap replications.
